# Supplementary material for: Content-rich biological network constructed by mining PubMed abstracts
Source: BMC Bioinformatics. 2004 Oct 8;5:147. doi: 10.1186/1471-2105-5-147 (PMC528731; doi:10.1186/1471-2105-5-147)
Supplement: Additional File 2 — The original results of the above study (non-essential files are deleted to keep the file size under the limit set by BMC bioinformatics). [file 1471-2105-5-147-S2.bz2 › chilibotAdditionalFile2/dip30/753ID9852089E18138/html/DNAA_DNAB.html]

 


 **DNAA** and **DNAB** 
  
Found 99 abstracts in PubMed, retrieved 30.  
 

 What does Google say? 
 PDF only 
| .edu only 

---

**Interactive relationship** (e.g. stimulation, inhibition, etc)

**Neutral relationship**- The interaction domains of the  **DnaA**  and  **DnaB**  replication proteins of Escherichia coli.  Ref: 10972842 Mol Microbiol, 2000
- Escherichia coli  **DnaA**  protein loads a single  **DnaB**  helicase at a  **DnaA**  box hairpin.  Ref: 12161435 J Biol Chem, 2002
- We propose that contacts between  **DnaA**  and  **DnaB**  occur via two interaction sites on each of the proteins.  Ref: 10972842 Mol Microbiol, 2000
- Both  **DnaA**  and TrfA proteins are required for  **DnaB**  induced template unwinding.  Ref: 9407123 J Biol Chem, 1997
- We show that  **DnaA**  protein actively mediates the entry of  **DnaB**  at oriC.  Ref: 9852089 J Biol Chem, 1998
- Other evidence suggests that proline 28 of  **DnaA**  is involved in the recruitment of  **DnaB**  to oriC.  Ref: 12864864 Mol Microbiol, 2003
- Although the  **DnaA**  protein directs the  **DnaB**  DnaC complex to the plasmid replication origin, it cannot by itself activate the helicase.  Ref: 9407123 J Biol Chem, 1997
- With this system, a specific interaction between the  **DnaA**  and  **DnaB**  proteins is required for delivery of the helicase to the RK2 origin region.  Ref: 9407123 J Biol Chem, 1997
- Domains of  **DnaA**  protein involved in interaction with  **DnaB**  protein, and in unwinding the Escherichia coli chromosomal origin.  Ref: 8702501 J Biol Chem, 1996
- These studies suggest regions of  **DnaA**  protein involved in interaction with  **DnaB**  protein, and in unwinding of oriC, or low affinity binding of ATP.  Ref: 8702501 J Biol Chem, 1996
- We defined physical contacts between  **DnaA**  and  **DnaB**  that involve residues 24 86 and 130 148 of  **DnaA**  and residues 154 210 and 1 156 of  **DnaB**  respectively.  Ref: 10972842 Mol Microbiol, 2000
- Size exclusion chromatography, surface plasmon resonance, and electron microscopy experiments demonstrated that the  **DnaB**  helicase is delivered to the  **DnaA**  box region, which is localized approximately 200 base pairs upstream from the region of origin opening and a potential site for helicase entry.  Ref: 11316803 J Biol Chem, 2001
- **DnaA**  was distributed throughout the cytoplasm, but both  **DnaB**  and DnaI were always detected as foci during the cell division cycle.  Ref: 10844689 Mol Microbiol, 2000
- the  **DnaA**  boxes must be on the correct side of the helix to load  **DnaB**  helicase.  Ref: 9525957 J Biol Chem, 1998
- That only two  **DnaB**  hexamers are bound, one for each replication fork, suggests that the binding of additional molecules of  **DnaA**  in forming the prepriming complex restricts the loading of additional  **DnaB**  hexamers that can bind at oriC.  Ref: 11551962 J Biol Chem, 2001
- The addition of  **DnaA**  extends melting to the AT rich region, which could then serve as the entry site for the  **DnaB**   **DnaA**  complex, much as has been documented for oriC dependent replication.  Ref: 9003285 Mol Gen Genet, 1996
- The initiation of chromosome replication in Escherichia coli requires the recruitment of the replicative helicase  **DnaB**  from the DnaBC complex to the unwound region within the replication origin oriC, supported by the oriC bound initiator protein  **DnaA** .  Ref: 10972842 Mol Microbiol, 2000
- The  **DnaA**  replication initiation protein has been shown to be essential for DNA strand opening at the AT rich region of the replication origin of the Escherichia coli chromosome as well as serving to recruit and position the  **DnaB**  replicative helicase at this open region.  Ref: 12835421 Proc Natl Acad Sci U S A< MedlineTA>Proc Natl Acad Sci U S A, 2000

**Non-interactive relationship** (e.g. studied together, co-existance, homology, etc.)

- Thus, the TrfA 44 initiation protein has the multifunctional properties of recruiting and positioning an active form of the  **DnaB**  helicase at the RK2 replication origin by a  **DnaA**  independent process.  Ref: 12835421 Proc Natl Acad Sci U S A< MedlineTA>Proc Natl Acad Sci U S A, 2000
- Similar stabilization of O2 Co II Blm bound to  **DNAa**  but not to  **DNAb**  was also observed by ESR spectroscopy.  Ref: 9100034 Biochemistry, 1997
- We report here that the Escherichia coli replication proteins  **DnaA** , which is required to initiate replication of both the chromosome and plasmid pSC101, and  **DnaB** , the helicase that unwinds strands during DNA replication, have effects on plasmid partitioning that are distinct from their functions in promoting plasmid DNA replication.  Ref: 10601213 J Bacteriol, 1999
- Co and Fe bleomycins Blms have been reacted with  **DNAa** , d GGAAGCTTCC 2, containing a specific site for cleavage, and  **DNAb** , d GGAAATTTCC 2, a closely related nonspecific 10 mer, to survey whether features of structure and reactivity of these adducts vary systematically as a function of the base sequence of the DNA oligomer.  Ref: 9100034 Biochemistry, 1997
- In the prepriming complex, the stoichiometries are 10  **DnaA**  monomers and 2  **DnaB**  hexamers per oriC plasmid.  Ref: 11551962 J Biol Chem, 2001
- Since  **DnaA**  induces a local unwinding of oriC, one model is that the limited availability of single stranded DNA at oriC restricts the number of  **DnaB**  molecules that can bind.  Ref: 12161435 J Biol Chem, 2002
- The Escherichia coli PriA protein loads the  **DnaB**  replicative helicase at branched DNA structures independently of the replication initiator protein,  **DnaA** , and thereby facilitates assembly of functional replisomes at sites removed from oriC.  Ref: 12581361 Mol Microbiol, 2003
- However, as shown in this study, TrfA 44 specifically interacts with the  **DnaB**  replicative helicase of Pseudomonas Pseudomonas putida and Pseudomonas aeruginosa and initiates the formation of a prepriming open complex in the absence of  **DnaA**  protein.  Ref: 12835421 Proc Natl Acad Sci U S A< MedlineTA>Proc Natl Acad Sci U S A, 2003
- In E.coli DnaT and DnaC are responsible for loading  **DnaB**  onto the DNA during primosome assembly, in the PriA and  **DnaA**  mediated cascades, respectively.  Ref: 11842108 Nucleic Acids Res, 2002
- Stoichiometry of  **DnaA**  and  **DnaB**  protein in initiation at the Escherichia coli chromosomal origin.  Ref: 11551962 J Biol Chem, 2001
- The AT rich region could be unwound by E. coli  **DnaA**  protein, and the  **DnaB**  helicase was loaded into the single stranded bubble.  Ref: 11606418 EMBO Rep, 2001
- This is based on the competition between the lambda P protein and the host  **DnaA**  and DnaC proteins for  **DnaB**  helicase.  Ref: 11429468 Microbiology, 2001
- As in the case of several Gram positive bacteria, essential genes involved in basic cellular functions, such as DNA or RNA metabolism  **dnaA** ,  **dnaB** , dnaN, gyrB, gyrA, pcnB, recF, rnpA, ssb, cell wall synthesis ponA, pbpA and probably cell division gidB, rodA were found.  Ref: 8969512 Microbiology, 1996
- Although the origin opening caused by binding of the host  **DnaA**  protein has been shown to be critical for the loading of the  **DnaB**  helicase, to date there has been no direct evidence presented for the formation of the  **DnaB**  complex at the  **DnaA**  box site.  Ref: 11316803 J Biol Chem, 2001
- Strand specific loading of  **DnaB**  helicase by  **DnaA**  to a substrate mimicking unwound oriC.  Ref: 12421318 Mol Microbiol, 2002
- High levels of  **DnaA**  protein in the presence of the TrfA protein also resulted in the stimulation of open complex formation and  **DnaB**  helicase loading on oriV, even in the absence of the four  **DnaA**  boxes.  Ref: 10417640 Mol Microbiol, 1999
- As was observed previously for  **DnaA** , overexpression of the wild type  **DnaB**  protein conversely stabilized the inheritance of partition defective plasmids while not increasing plasmid copy number.  Ref: 10601213 J Bacteriol, 1999
- Although, interaction of  **DnaA**  with  **DnaB**  could not substitute for RepA  **DnaB**  interaction for helicase loading,  **DnaA**  along with integration host factor, DnaC, and RepA was essential for helicase loading.  Ref: 9874774 Proc Natl Acad Sci U S A, 1999
- The initiation of replication from oriV RSF1010, the replication origin of the broad host range plasmid RSF1010, depends on RepA helicase, RepB primase, and RepC initiator protein, encoded by RSF1010 itself, while this initiation event in E. coli is independent of  **dnaA** ,  **dnaB** , dnaC, and dnaG Scherzinger et al.  Ref: 8690079 FEBS Lett, 1996
- One can also conclude that the  **DnaA**  regulated transcriptional activation of ori lambda acts at the step, in which all these gene products cooperate, i.e. during preprimosome loading and chaperone mediated release of  **DnaB**  from P protein inhibition.  Ref: 8914519 Mol Gen Genet, 1996
